# Convolutional neural network approach for automated well zonation in the Lower Bahariya member north Western Desert Egypt

**DOI:** 10.1038/s41598-025-32119-2

**Published:** 2025-12-26

**Authors:** Khaled Saleh, Walid M. Mabrouk, Ahmed M. Metwally

**Affiliations:** 1 Department of Geophysics, Faculty of Science, Cairo University, Giza, 12613 Egypt; 2 PetroShahd Company, Zahraa Maadi, Cairo, Egypt

**Keywords:** Well correlation, Zonation, Convolutional neural network, Lower Bahariya, Mathematics and computing, Solid Earth sciences

## Abstract

Accurate well-to-well stratigraphic zonation is fundamental to subsurface reservoir characterization, particularly in geologically complex settings such as tidal channel systems where lithological variability and discontinuous shale barriers pose significant interpretation challenges. This study introduces a novel, image-based workflow leveraging Convolutional Neural Networks (CNNs) to automate zonation in newly drilled wells using conventional well log data. The proposed approach transforms multiple input features—including Gamma Ray (GR), Density (RHOB), Neutron Porosity (NPHI), Photoelectric Effect (PE), interpreted facies, True Vertical Depth Subsea (TVDSS), and three spatial distance features—into images. Each image represents a normalized vertical window of subsurface data and is labeled according to expert-interpreted stratigraphic zones. These labeled images form the training dataset for a supervised CNN classification model. The methodology is applied to the Shahd SE field in the northern Western Desert of Egypt, targeting the Lower Bahariya member. The trained CNN model is evaluated on blind wells demonstrating high prediction accuracy and successful generalization across wells. The proposed workflow introduces a novel image-based transformation of 1D well-log sequences into 2D representations that enables the use of convolutional neural networks traditionally designed for spatial image analysis. Unlike previous zonation studies that rely on raw 1D signals or statistical clustering, our approach (i) applies an optimized pseudo-image encoding of log data, (ii) integrates inter-well spatial distance as an explicit feature to enhance geological continuity, and (iii) incorporates post-processing filtering to refine zone boundaries. This combination provides a more robust and automated zonation methodology with improved generalization across blind wells.

## Introduction

Well-log correlation is a fundamental step in subsurface geological interpretation and reservoir characterization. It involves aligning lithological boundaries across multiple wells to construct a continuous stratigraphic framework, essential for geological modeling and predicting lateral variations in lithology^1,2^. This step is particularly critical in heterogeneous depositional settings, such as fluvial and tidal systems, where rapid facies changes and discontinuous shale barriers can significantly influence reservoir connectivity and fluid flow behavior. In both static and dynamic reservoir models, accurate correlation directly impacts reservoir compartmentalization and the reliability of simulation^3,4^.

In the Shahd SE Field, one of the key challenges lies in establishing reliable well-to-well correlations within the Lower Bahariya member. This formation exhibits a complex architecture, composed of multiple stacked sand channels that often share similar petrophysical log responses—such as gamma ray, density, and porosity—making it difficult to delineate individual zones using traditional manual methods^5^. The challenge is further intensified by the presence of thin or laterally discontinuous shale barriers, which are difficult to resolve yet critically control fluid flow and compartmentalization. Misinterpretation of these features can lead to overestimated connectivity and poor history matching during reservoir simulation. Recent studies have highlighted the significant influence of facies and diagenesis on reservoir heterogeneity, emphasizing the importance of developing data-driven zonation approaches that incorporate lithological and diagenetic characteristics^6,7^.

Traditional well correlation methods are often subjective, labour-intensive, and prone to inconsistency, especially in geologically complex or data-sparse environments^1^. These limitations have motivated the development of automated, data-driven solutions leveraging machine learning and deep learning techniques. Early work by Luthi and Bryant (1997) demonstrated the ability of neural networks to improve the consistency of stratigraphic markers across wells^8^. More recent advances include attention-based convolutional neural networks (CNNs), which can automatically learn lithological patterns from log data and accurately predict stratigraphic boundaries^9^. Unsupervised approaches—such as Principal Component Analysis PCA and wavelet transforms combined with dynamic time warping (DTW)—have also been employed to identify facies and improve zone correlations in complex depositional^10,11^. Broader evaluations by Ma et al. (2011) and recent findings by Zhang et al. (2024) confirm that deep learning models trained on expert-annotated datasets outperform conventional methods, reducing interpretation bias and enhancing geological continuity^12,13^.

Building on this foundation, the present study proposes an automated CNN-based zonation workflow tailored for the Shahd SE Field. The methodology leverages the spatial variation of well log signatures—driven by lateral and vertical heterogeneities in depositional environments—along with depth and location-based features, to identify and correlate stratigraphic zones without manual intervention. The aim is to enhance the efficiency, objectivity, and geological consistency of the correlation process.

In contrast to traditional manual and semi-automated methods, the proposed CNN-based approach provides a quantitative assessment of correlation accuracy, enabling objective evaluation through statistical metrics such as classification accuracy and zone match percentage. This ensures reproducible and data-driven correlations that outperform subjective interpretations in both consistency and precision.

This study advances automated well zonation by introducing a workflow that differs fundamentally from previous CNN-based or machine-learning zonation approaches. First, we transform 1D well-log sequences into optimized 2D pseudo-images, allowing 2D CNNs to extract spatially invariant depth patterns that are difficult for 1D models to capture. Second, the workflow incorporates inter-well spatial distance as an additional input feature, enabling the model to account for lateral heterogeneity and geological continuity—an aspect rarely included in earlier zonation studies. Third, a set of post-processing filters is applied to stabilize zone transitions and remove noise-driven fluctuations. Together, these components constitute a unique, integrated framework that enhances the accuracy, stability, and transferability of automated zonation.

## Geological background

**Fig. 1:**
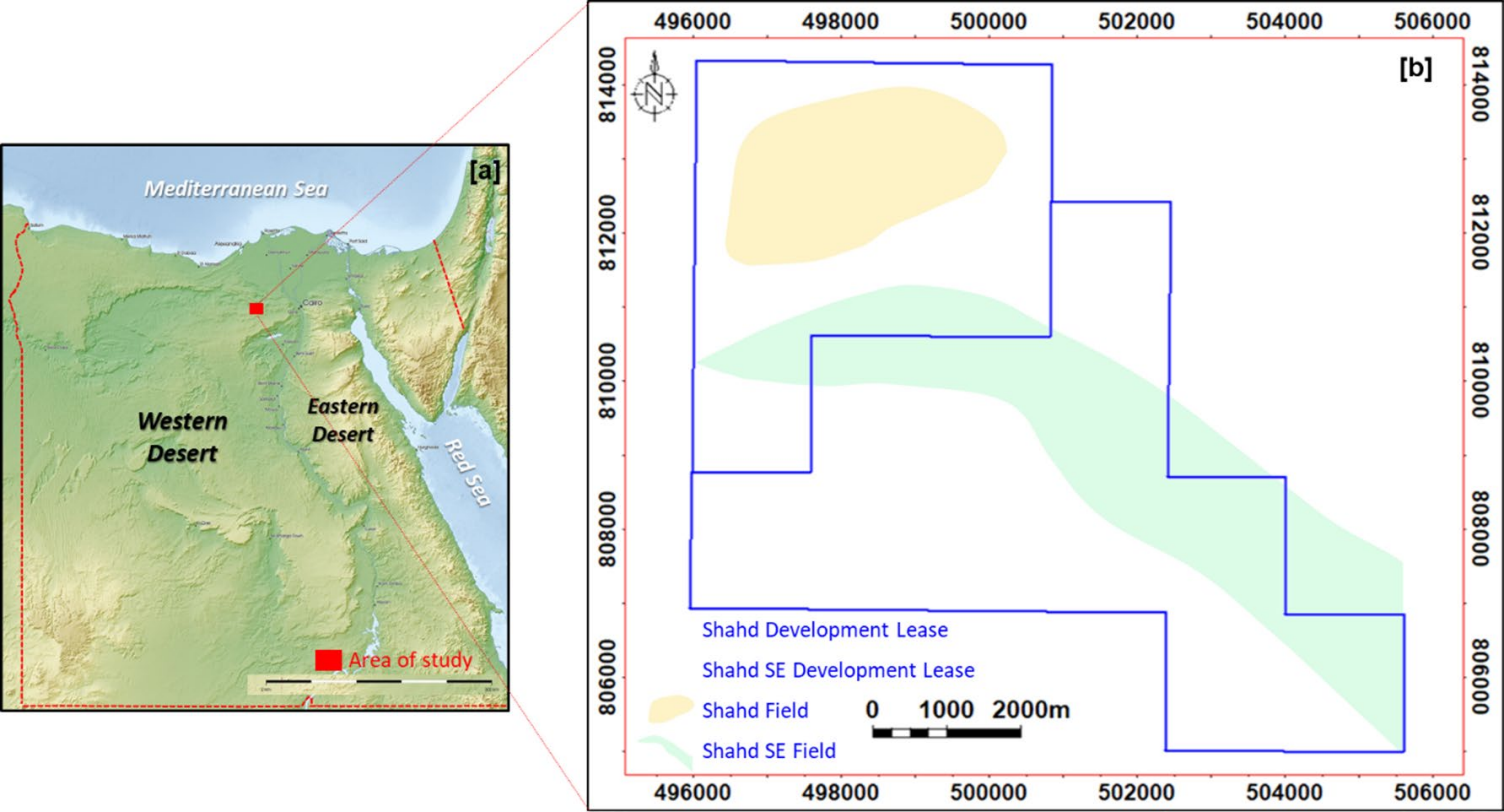
Location map of the Shahd SE Field in the northern Western Desert of Egypt (Modified after mapswire, https://mapswire.com/maps/egypt/). Panel (b) illustrates the Shahd and Shahd SE Development Leases, Shahd SE Development Lease, and the outlines of the Shahd and Shahd SE Fields (Generated using Petrel 2024.6, https://www.software.slb.com/products/petrel).

The Shahd SE Field is located in the northeastern part of Egypt’s Western Desert and is operated by PetroShahd (Fig. 1). Discovered in 2007, the field targets Cenomanian-age reservoirs, specifically the Upper and Lower Bahariya members. The lithological makeup of the Bahariya Formation is vertically heterogeneous. The upper part comprises intercalated thin-bedded clastics—primarily shale, siltstone, and sandstone—with occasional carbonate streaks, whereas the lower section is dominated by stacked, relatively clean sandstone channels separated by shale barriers of variable continuity. Structurally, the field is defined by WNW-ESE trending fault-bounded closures, which influence reservoir distribution and compartmentalization.

The primary reservoir rock is sandstone with an average porosity of around 20% within the Lower Bahariya member^14^. Approximately 40 wells have been drilled in the field, with development still ongoing. Facies analysis and depositional environment interpretation using formation micro imager log (FMI) study over Shahd SE field provided detailed information about the depositional environments and the reservoir geometry^15^. The study concluded that the Lower Bahariya member in the studied wells was interpreted as a tidal depositional environment represented by the following sub-environments: tidal channels, tidal bars/tidal delta and tidal flat sand-mud. The study subdivided the clean sand part of the Lower Bahariya member (excluded the upper part) into six individual sedimentological units were defined based on detailed description of sedimentary structures for Shahd SE-5 well from the image logs described in Table 1.

**Table 1:** Summary of zonation units from the formation Micro-Imager (FMI) study within the Lower Bahariya member for Shahd SE-5.

Unit	Thickness	Description
Unit 6	28 ft.	Thin laminated sandstone ended by short cycles of coarsening upward tidal bars with internal flaser bedding indicating tidal flat environment
Unit 5	36 ft.	Consists of massive to faintly laminated with some fining upward ripple sandstone and ended by coarsening upward cycle of forest tabular cross bedding suggesting tidal bars and/or tidal delta
Unit 4	10 ft.	Thin laminated hetrolithic bioturbated facies with dispersed pyrite and directly deposited over the underlying tidal channels
Unit 3	43 ft.	Consists of two fining upward cycles interbeded with small coarsening upward cycle (tidal bar or delta)
Unit 2	59 ft.	Consists of upward of laminated, small rippled sandstone with some transgressive mudstone
Unit 1		Tidal flat deposits forms the bottom of Lower Bahariya member and it is unconformably overlying the Khartia Formation

Another comprehensive biostratigraphic and sedimentological analysis was carried out for the Lower Bahariya reservoir across the Shahd and Shahd SE structures^16^. This study involved a cyclo-chronostratigraphic interpretation of thirteen wells, resulting in the subdivision of the Lower Bahariya sequence into eight distinct sedimentary cycles. The boundaries of these cycles and sub-cycles were defined based on depositional breaks and truncations, as indicated by Pattern Recognition of Events by Frequency Analysis (PEFA) patterns and Integrated Pattern Recognition of Events by Frequency Analysis (INPEFA) curves. This high-resolution stratigraphic framework enabled a detailed understanding of sand distribution and facies variability within each cycle. The identified cycles, designated from top to bottom as A, B, C, D, E, F, G, and BBC, are summarized in Table 2 along with their respective thicknesses and depositional characteristics.

**Table 2:** Summary of zonation units from the biostratigraphic and sedimentological study within the Lower Bahariya member.

Cycle	Thickness	Description
A	60–70 ft.	Carbonate-dominated cycle that marks the end of the Lower Bahariya member deposition at Shahd and Shahd SE structures. It developed under a shallow marine open system setting
B	10–40 ft.	Glauconitic sandstone facies representing a shallow shelf environment. This cycle is absent in the central crestal area due to erosion during the deposition of Cycle D
C	25–50 ft.	Developed at well Shahd SE-1 in the south eastern part of Shahd SE field, this cycle is characterized by shoreface to offshore facies
D	30–70 ft.	Sandstone-dominated cycle representing the peak development of the barrier system. It extends across much of the central area, with the bar crest flanked by marine facies
E	40–100 ft.	Dominated by sandy barrier bar facies, this cycle forms two main clusters around Shahd SE-5 and Shahd SE-2 wells (bar crest facies). These are laterally surrounded by bar margin facies that grade southeastward into lower shoreface to offshore deposits
F	5–20 ft.	Comprises two localized depositional lobes identified at Shahd-2ST, Shahd-3, Shahd SE-5, and Shahd SE-8. These bodies truncate the crestal facies of the main barrier bar complex (BBC)
G	5–15 ft.	A locally identified sandstone/shale cycle with lower shoreface to offshore facies. It is only observed incised into the BBC at Shahd-2ST and not recognized in other wells
BBC	30–70 ft.	NW-SE trending shallow marine barrier bar system

The biostratigraphic, sedimentological, and Formation MicroImager (FMI) studies form the fundamental basis for well-to-well correlation. In this study, insights from both sources were integrated into a unified zonation scheme, as summarized in Table 3. It is worth noting that Cycle B, as defined in the biostratigraphic study, is localized in Shahd field and not present within the current study area. Additionally, the FMI study did not provide a description for the Cycle A. For wells not originally included in the biostratigraphic or FMI analyses, additional zonation interpretations were conducted by domain experts. These interpretations followed the same criteria and log response patterns established in the original studies, ensuring consistency and enhancing the overall quality and performance of the model.

**Table 3:** Integrated stratigraphic zonation framework used in this study, aligning the derived zones with formation Micro-Imager (FMI) units and corresponding sedimentological cycles.

Current study (zone)	Mapped FMI unit (s)	Mapped sedimento-logical cycle(s)	Description
Cycle A	–	Cycle A	Carbonate-dominated cycle that marks the end of Lower Bahariya deposition. Formed under shallow marine open system conditions
Upper Sand Unit (USU)	Units 6 & 5	Cycles C & D	Uppermost sandstone channel. Thin laminated to cross-bedded sandstones (tidal bars/shoreface bars), showing coarsening-upward trends and internal flaser bedding. Represents bar crest sands deposited at peak barrier development
Shale Barrier 1	Unit 4		Thin laminated heterolithic bioturbated facies with dispersed pyrite, interpreted as lower shoreface or lagoonal muds forming a muddy barrier between USU and MSU
Middle Sand Unit (MSU)	Unit 3	Cycles E & F	Middle sandstone channel. Stacked fining-upward and coarsening-upward cycles (tidal bar or delta), representing barrier bar clusters and depositional lobes
Shale Barrier 2			Bar-margin mudstones and heteroliths that grade seaward into lower-shoreface facies; act as a shale barrier separating MSU from LSU
Lower Sand Unit (LSU)	Units 2	Cycle G & BBC	Laminated and rippled sandstone with some mudstone. Represents a NW–SE trending barrier bar system (BBC)
Tidal Flat Base	Unit 1		Basal tidal flat mudstone/siltstone that unconformably overlies the Khartia Formation, acting as a lower seal

**Fig. 2:**
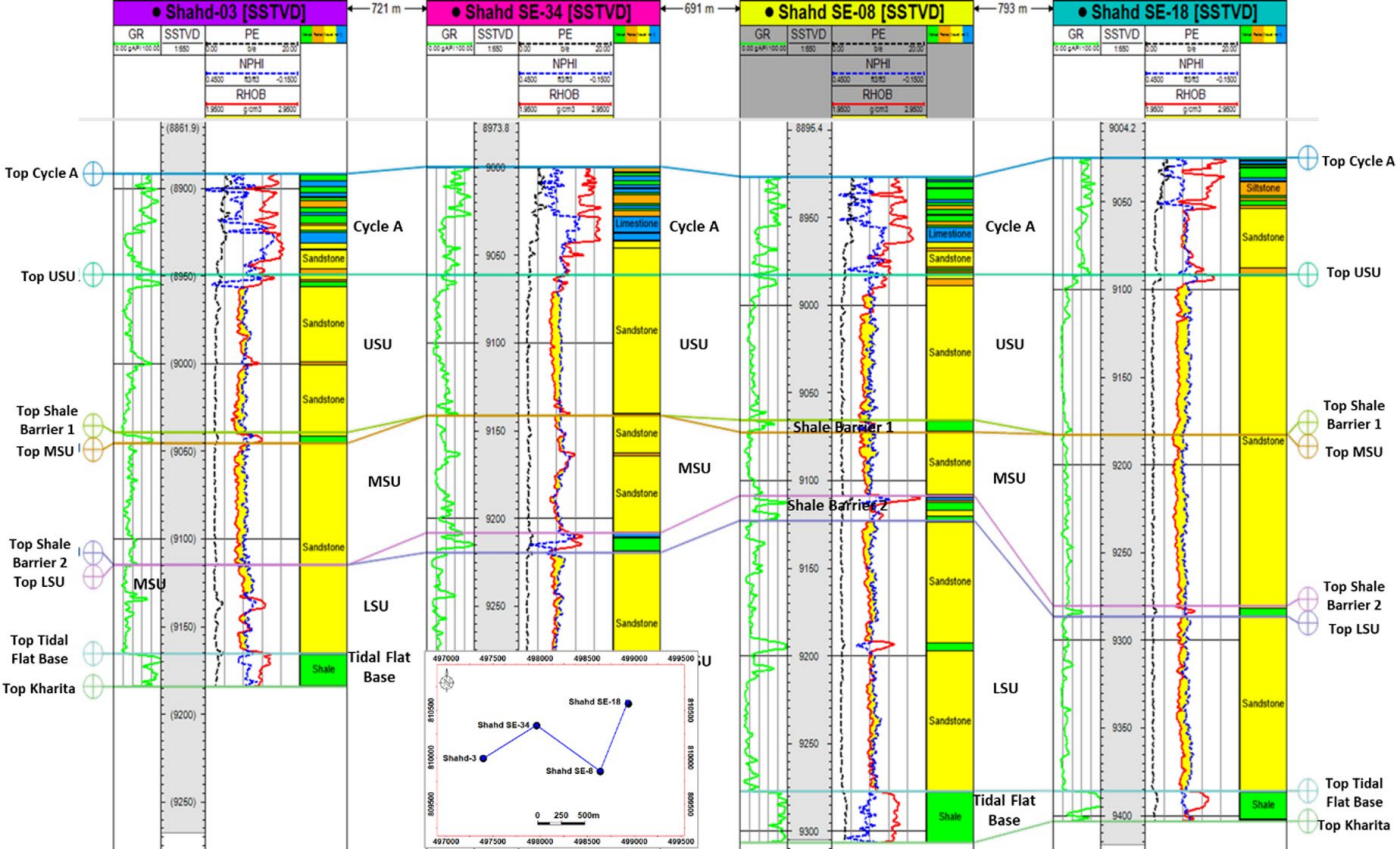
Stratigraphic correlation panel of four Shahd SE wells showing the internal subdivision of the Lower Bahariya member into seven stratigraphic zones.

Figure 2 presents a stratigraphic correlation panel across four wells (Shahd-03, Shahd SE-34, Shahd SE-08, and Shahd SE-18) within the Lower Bahariya member. The correlation highlights the subdivision of the member into seven discrete stratigraphic zones: Cycle A, Upper Sand Unit (USU), Shale Barrier 1, Middle Sand Unit (MSU), Shale Barrier 2, Lower Sand Unit (LSU), and the Tidal Flat Base. These zones were identified based on integrated log signatures—particularly Gamma Ray (*GR*), Neutron Porosity (*NPHI*), Density (*RHOB*), and Photoelectric (*PE*) curves—alongside lithological and facies interpretations.

Table 4 summarizes the observed minimum and maximum thicknesses for each interpreted zone based on expert geological correlation and well log analysis. These thickness intervals not only capture the typical range of vertical deposition but also serve as essential constraints for downstream machine learning applications. Specifically, they are later used as filtering thresholds during the post-processing of model predictions to exclude stratigraphic intervals that fall outside the known geological thickness range, thereby enhancing the plausibility of automated zonation results.

**Table 4:** Minimum and maximum thickness values for each stratigraphic zone derived from the training dataset.

Zone	Minimum thickness (ft.)	Maximum thickness (ft.)
Cycle A	55.5	79.5
Upper Sand Unit (USU)	51.5	85.0
Shale Barrier 1	2.0	13.5
Middle Sand Unit (MSU)	17.5	93.0
Shale Barrier 2	8.0	37.0
Lower San Unit (LSU)	38.5	73.0
Tidal flat base	4.5	23.0

## Methodology

**Fig. 3:**
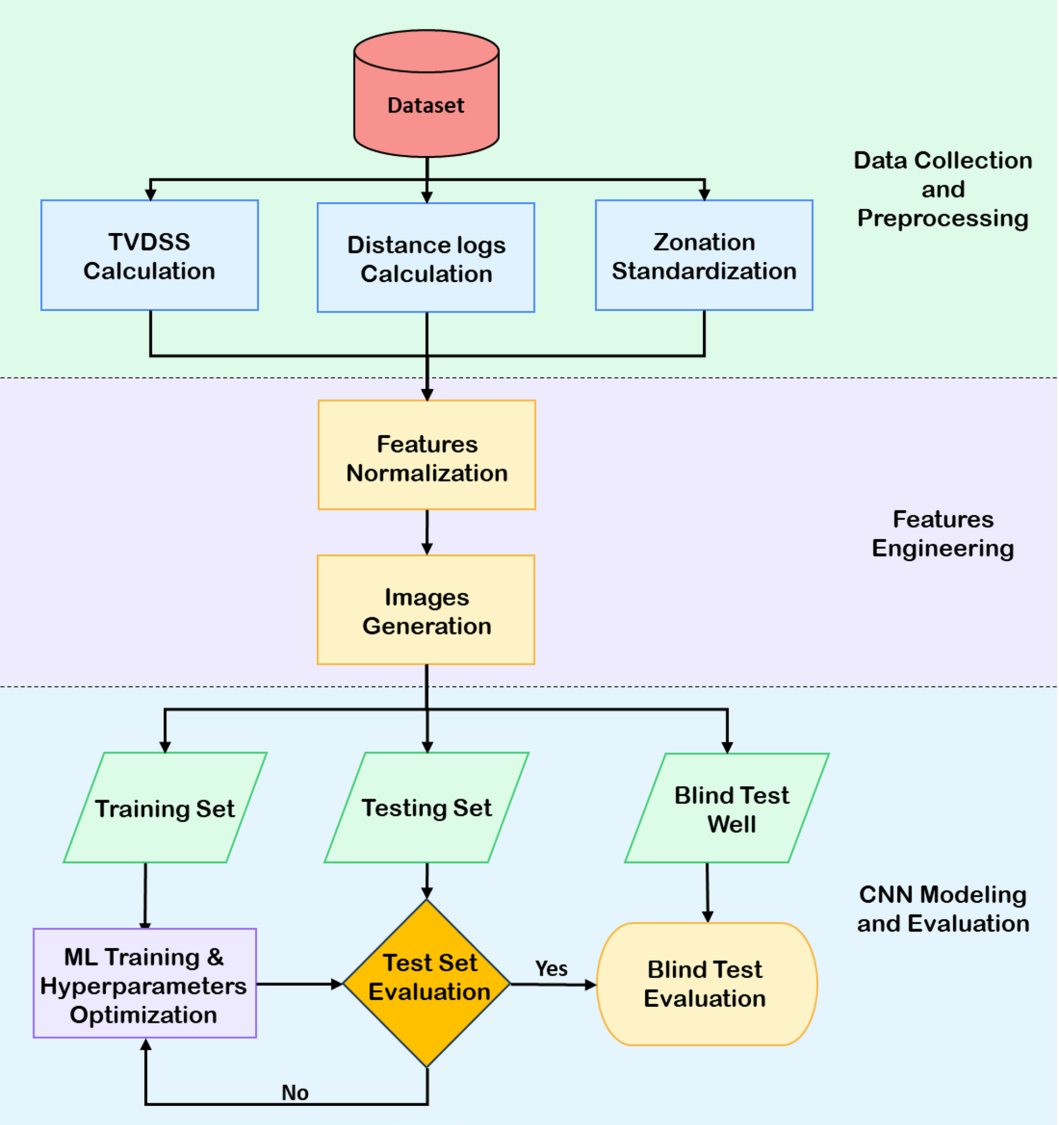
Summarized workflow for automated zonation prediction using CNNs. The process includes data preprocessing, feature engineering, image generation, dataset splitting, model training with hyperparameter tuning, and evaluation.

This study aims to design an automated workflow that leverages a convolutional neural networks (CNNs) to perform stratigraphic zonation of the Lower Bahariya reservoir in the Shahd SE field, located in the north of Egypt’s Western Desert. Figure 3 displays the proposed workflow, which begins with the collection and integration of training data. This dataset includes conventional wireline logs (e.g., *GR*,* RHOB*,* NPHI* and *PE*), True Vertical Depth below Subsea (*TVDSS*), interpreted facies, and pre-defined zonation for each well.

As part of the preprocessing stage, the lateral spatial relationship between wells is quantified by calculating the distance of each well from three reference points. This step is essential for capturing lateral variability in facies distributions due to structural and depositional heterogeneity across the field.

Following preprocessing, the input features are normalized using appropriate scaling strategies tailored to the distribution and range of each feature, ensuring comparability and promoting effective model convergence. The normalized features are then transformed into 2D image-like representations, where each sample is labelled according to its corresponding stratigraphic zone. This image-based transformation enables the use of a convolutional neural networks (CNNs) to exploit spatial patterns in the data.

The dataset comprises 22 training wells, each characterized by nine input features. The data is partitioned into training and testing subsets to facilitate CNN model training and hyperparameter tuning, ensuring the model achieves optimal performance and generalizes well to unseen data.

Finally, the trained model is evaluated on a blind test well—completely excluded from the training process—as a final validation step. This evaluation assesses the model’s predictive capability and robustness under realistic application conditions, particularly its effectiveness in stratigraphic zonation for newly drilled wells.

### Data collection and preprocessing

The preparation of data include the collection of the wireline logs that include the gamma ray log (*GR*), Density log (*RHOB*), Neutron porosity log (*NPHI*) and photoelectric log (*PE*). In addition, facies interpretation log (*Facies*) were used as the recognizing depositional heterogeneities is essential for defining flow units and guiding reservoir development^17^. Although facies logs are inherently interpretive, their inclusion as categorical features in the CNN-based workflow serves to enhance stratigraphic and lithological context rather than impose interpreter bias.

An additional input feature incorporated into the model is the True Vertical Depth below Subsea (*TVDSS*), calculated from the well’s deviation survey using the Minimum Curvature Method to accurately represent the vertical position of subsurface formations.

In sequence stratigraphy, the concept of stratigraphic order is fundamental—sedimentary layers are deposited in a predictable vertical sequence governed by changes in relative sea level, accommodation space, and sediment supply^18,19^. Incorporating TVDSS into the model enables it to capture this stratigraphic ordering, helping the CNN learn the relative positioning of zones within a consistent vertical framework. This enhances the model’s ability to distinguish between stratigraphic boundaries and supports more reliable zonation predictions.

An important factor to account for in reservoir zonation is the spatial location of each well, as depositional facies often exhibit lateral variations even within the same stratigraphic unit and depositional environment. These lateral changes are common in heterogeneous systems such as shoreface, fluvial, or tidal deposits, where sedimentary structures and lithofacies can vary significantly over short distances while still representing the same genetic depositional cycle^20,21^.

**Fig. 4:**
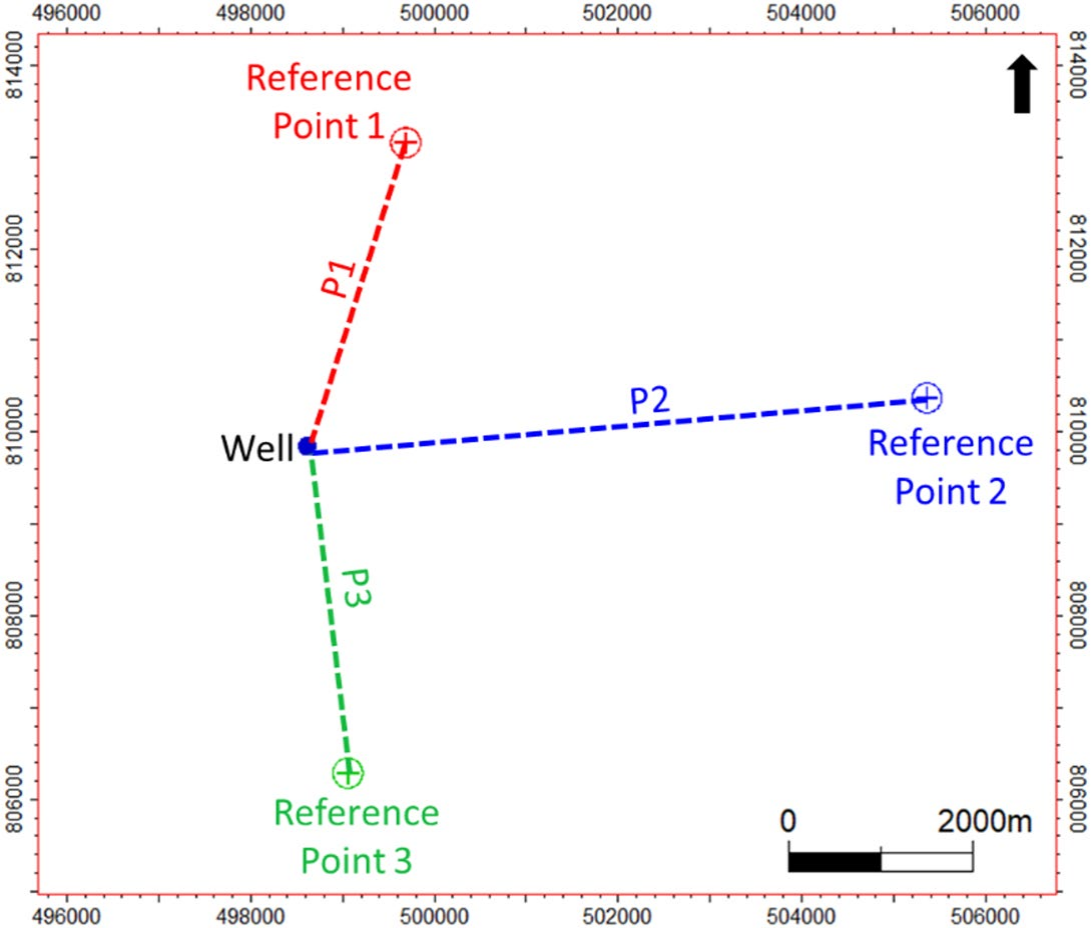
Example illustration of the directional distance features (P1, P2, and P3) used in the model.

To incorporate this lateral variability into the model, a novel approach was implemented by introducing three spatial distance-based features. These features represent the distances of each well from three geologically meaningful reference points that geometrically encompass the study area (Fig. 4). Since three distances are the minimum required to uniquely define a point in two-dimensional space, this method effectively captures the spatial position of each well within the reservoir.

Furthermore, these distances (*P1*,* P2* and *P3*) were calculated not solely based on surface coordinates but were also adjusted to account for well trajectory and deviation, thereby providing a more accurate representation of the true subsurface location of the data points used in training. This spatial awareness enhances the model’s ability to distinguish between lateral facies changes and genuine stratigraphic transitions, improving the reliability of the automated zonation.

To ensure data quality and consistency before feature engineering process, cutoff limits were applied to the measured logs to minimize the effect of outliers arising from instrumental or environmental factors such as washouts and borehole irregularities. The applied cutoff ranges were 0–150 API for gamma ray (GR), 1.95–2.95 g/cc for bulk density (ROHB), 0–0.45 for neutron porosity (NPHI), and 2–7 barns/e⁻ for photoelectric factor (PE). These thresholds represent the typical operational limits of reliable log responses and were used to eliminate noise and unrealistic measurements. The remaining features, including derived and interpreted inputs such as distance logs and categorical facies, were not subject to cutoff filtering since they were generated through controlled mathematical or interpretive processes that inherently maintain consistency.

### Features engineering

Feature engineering is a fundamental step in preparing the dataset for the automated zonation model. It begins with transforming the raw well data into a structured tabular format that includes all relevant input features and the associated target zones. The geological zonation is numerically encoded to correspond with predefined stratigraphic units, enabling supervised classification. Similarly, the facies log—originally described as Shale, Siltstone, Sandstone, and Carbonates—is mapped to integer values of 0, 1, 2, and 3, respectively.

Each selected feature serves a specific geological or spatial role. Conventional logs such as gamma ray (GR), density (RHOB), neutron porosity (NPHI), and photoelectric factor (PE) are retained due to their sensitivity to lithology and fluid content, making them effective indicators of stratigraphic variations and facies changes. In contrast, resistivity logs were excluded from the study, as they primarily reflect fluid properties rather than lithological variations, and thus are less suitable for the stratigraphic zonation objectives of this work^22,23^. The inclusion of True Vertical Depth Subsea (*TVDSS*) captures the vertical sequence of deposition, which is essential in stratigraphic interpretation. Additionally, three distance-based features (*P1*,* P2* and *P3*) are introduced to quantify the well’s spatial location relative to three fixed reference points (Fig. 4). These features allow the model to account for lateral depositional variability across the field, which is particularly important in heterogeneous environments like barrier bar and tidal systems.

In the normalization phase, a min-max scaling strategy is applied, tailored to the nature of each feature:

- For *TVDSS*,* GR*,* NPHI*,* RHOB* and *PE*, normalization is performed individually for each well to minimize discrepancies caused by logging tool variations and to ensure consistent intra-well feature representation.
- The facies log is normalized using a fixed scale of 0 to 3, consistent with its categorical encoding.
- The distance features (*P1*,* P2* and *P3*) are normalized across the entire dataset to retain the absolute spatial relationships between wells, thereby preserving critical lateral trends.

The subsequent step in the workflow is the conversion of the normalized well-log features into a format compatible with convolutional neural networks (CNNs) training. In this approach, each stratigraphic zone is represented not by a single 2D image combining all features, but by a set of nine separate 2D grayscale images, each corresponding to one of the input features.

Each feature-specific image has a fixed resolution of 64 rows × 8 columns. The 64 rows represent interpolated depth levels across the stratigraphic zone, effectively standardizing variable-thickness intervals to a common vertical resolution. The 8 columns are populated with repeated values of the corresponding normalized feature at each depth level. This design mimics a vertically aligned log curve “stretched” into a small rectangular image, allowing the CNN to detect vertical trends, transitions, and feature-specific patterns.

**Fig. 5:**
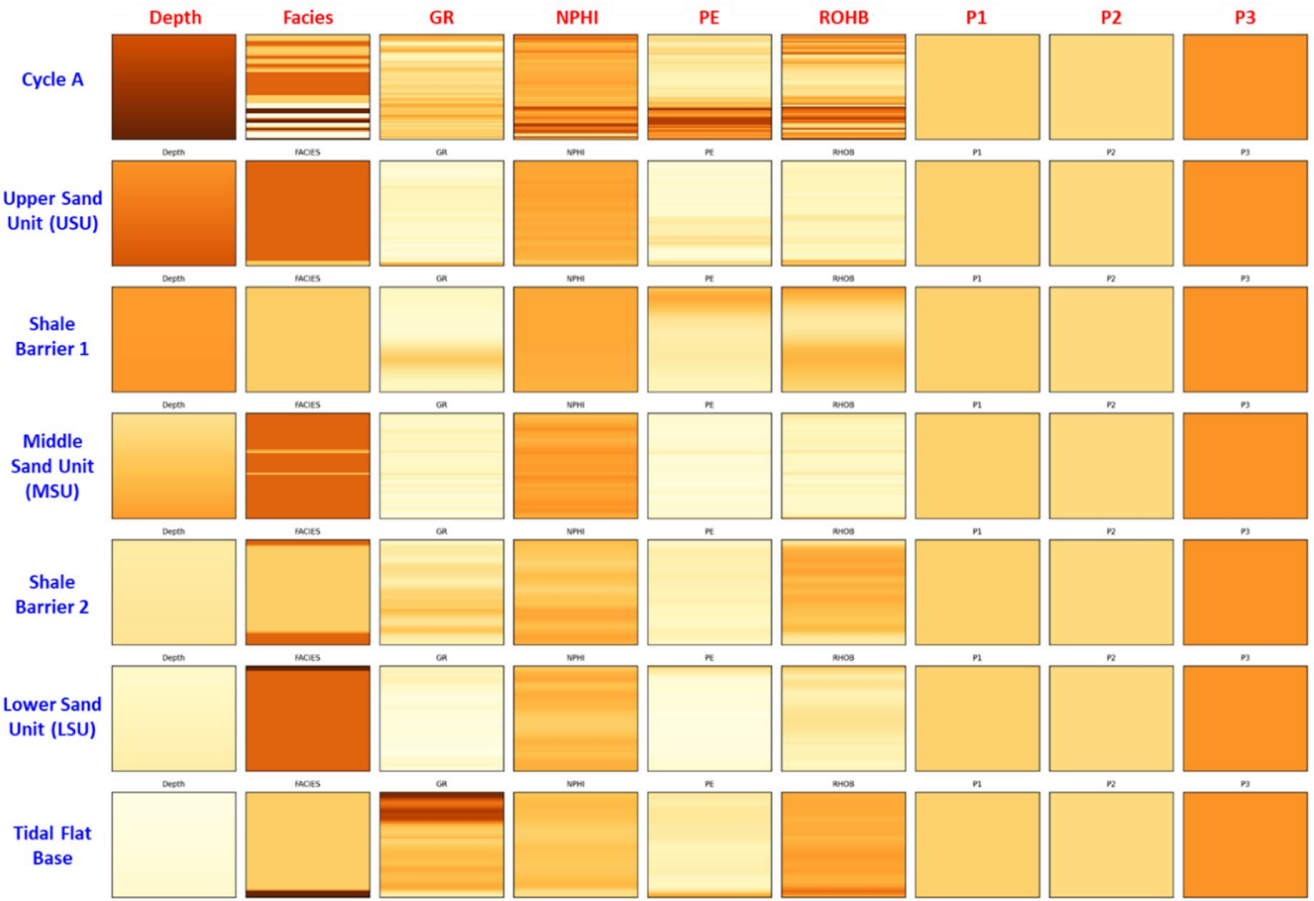
Image-wise representation of input features for a single well. Each row corresponds to a stratigraphic zone, and each column shows a 64 × 8 pixel image of a specific feature. Features include Depth, Facies, GR, NPHI, PE, RHOB, and distance attributes (P1, P2 and P3).

The result is a stack of 64 × 8 images—one for each feature—forming a 9-channel input tensor that encapsulates both depth-dependent log responses and lateral continuity within each feature domain (Fig. 5). Each of these stacked inputs is labeled with its corresponding stratigraphic zone, allowing the CNN model to learn feature-specific spatial patterns associated with different depositional zones.

By converting categorical facies data into image channels alongside continuous logs, the model learns joint spatial patterns and inter-feature relationships directly from the image pixels rather than assigning explicit numerical weights to categorical variables. Consequently, the CNN automatically determines the optimal weighting of each input through its learned convolutional kernels during training.

This representation enables the model to focus on vertical variation within each feature while maintaining a standardized spatial format suitable for training deep learning models. By treating each feature as an individual image channel, the CNN can learn complex, multi-feature interactions and stratigraphic signatures that support accurate zonation predictions across the field.

### Model training

The feature-engineered dataset was divided into a training set (80%) and a testing set (20%) to facilitate model training and internal validation. Additionally, a separate set of blind test wells—entirely excluded from the training and testing phases—was reserved to evaluate the model’s generalization capability on unseen data.

Although 1D convolutional neural networks (1D CNNs) are conventionally more suitable for sequential data such as well logs, preliminary experiments in this study revealed that the proposed 2D CNN architecture consistently outperformed an equivalent 1D CNN baseline in both prediction accuracy and generalization to blind wells. This improvement may stem from the way the 2D representation enables the model to exploit horizontal redundancy as a form of implicit regularization, smoothing noisy measurements along the synthetic horizontal dimension. The repeated columns can be viewed as multiple identical “views” that reinforce vertical structures, allowing the model to learn more robust filters. Moreover, 2D convolutions can capture spatially invariant depth patterns that remain stable under noise or slight misalignments—conditions that commonly affect well-log data.

Similar advantages of transforming one-dimensional time-series data into two-dimensional representations for convolutional processing have been demonstrated in prior studies. Hatami et al. converted time-series data into recurrence plots and reported superior performance of 2D CNNs compared to 1D architectures^24^. Likewise, recent research on sensor-based classification has shown that encoding multivariate time-series as 2D images can enhance feature discrimination and improve model accuracy^25^.

To automate stratigraphic zonation, a convolutional neural network (CNN) model was developed using 2D image representations of well log features. The input to the model consists of 64 × 8 single-channel grayscale images, where each row corresponds to normalized depth (vertical resolution), and each column reflects the normalized intensity of a specific petrophysical or spatial feature.

To determine the optimal image width for the 1D-to-2D transformation, an empirical optimization experiment was conducted. The model was trained and validated using different column repetitions (5, 6, 7, 8, 9, and 10), while keeping all other architectural parameters and hyperparameters constant. Among these configurations, the 8-column input provided the best trade-off between model accuracy and computational efficiency. It achieved the highest validation accuracy and fastest convergence rate, whereas larger widths (≥ 9) offered no additional improvement but increased training time. Conversely, smaller widths (≤ 6) reduced redundancy excessively and slightly degraded the model’s ability to generalize, suggesting that moderate horizontal repetition reinforces vertical pattern recognition and stabilizes the learning process.

**Fig. 6:**
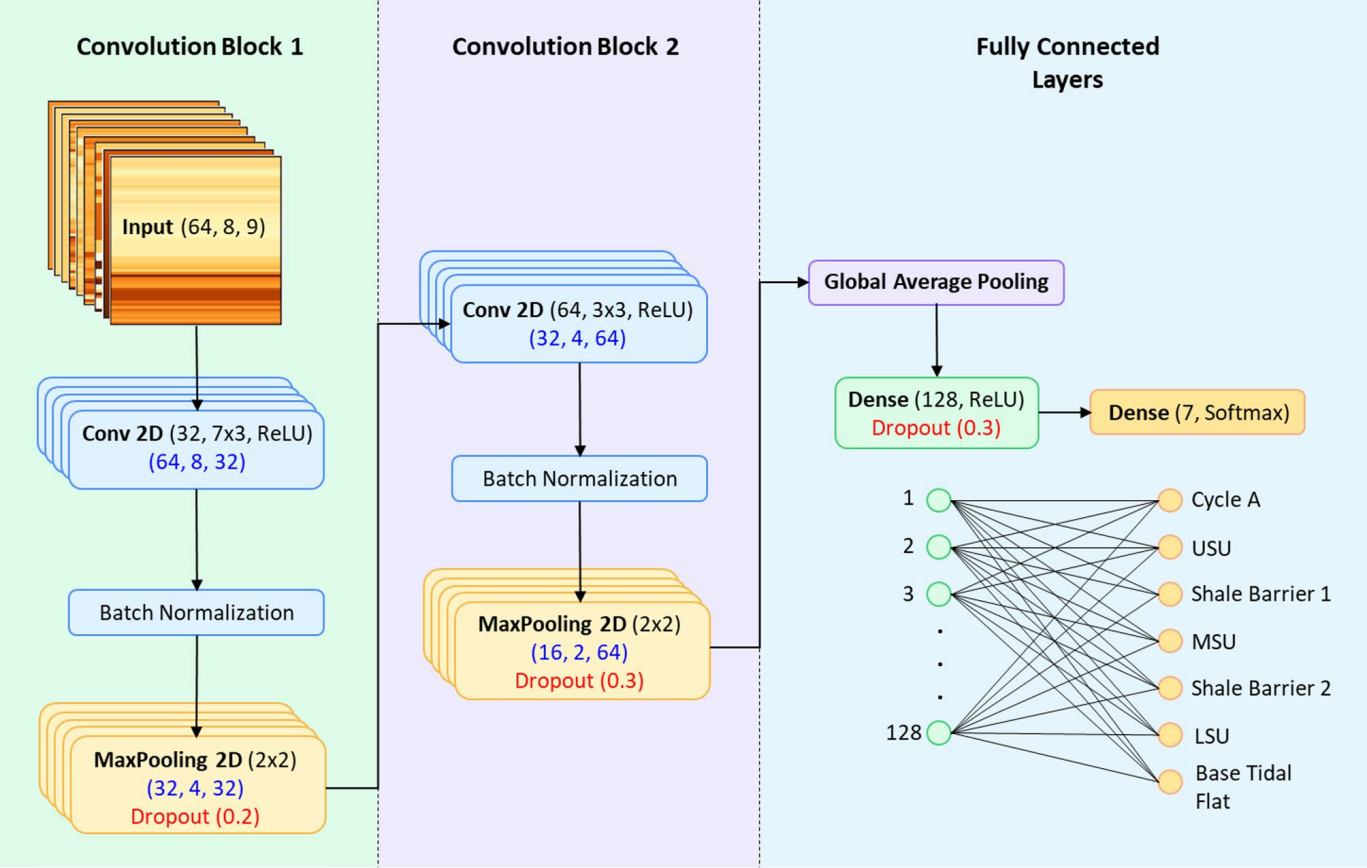
Architecture of the Convolutional Neural Network (CNN) model used for stratigraphic zone classification.

The architecture of the proposed Convolutional Neural Network (CNN) is designed to extract both spatial and hierarchical features from structured well-log image representations (Fig. 6). It comprises two primary convolutional blocks, each engineered to progressively capture complex patterns relevant to stratigraphic zonation.

The first convolutional block begins with a 2D convolutional layer that applies 32 filters with a kernel size of (7 × 3). This layer performs a spatial convolution over the input data, enabling the model to extract localized patterns across both depth and feature axes. The mathematical operation performed by the convolution layer is defined as:

$$\:{Y}_{i,j,k}=\sum\:_{m=1}^{M}\sum\:_{n=1}^{N}\sum\:_{c=1}^{C}{W}_{m,n,c,k}\:.\:\:{X}_{i+m,j+n,c}+\:{b}_{k}$$

Where $\:{Y}_{i,j,k}$ is the output feature map at location ($\:i,\:j$) in channel $\:{k}_{{\prime\:}}$, $\:{W}_{m,n,c,k}$ is the kernel weight of size $\:M\times\:N$ for the input channel $\:C$ and output channel $\:{k}_{{\prime\:}}$, $\:X$ is the input tensor and $\:{b}_{k}$ is the bias term.

This layer is followed by Batch Normalization, which standardizes the outputs to have zero mean and unit variance. This accelerates convergence and stabilizes learning by reducing internal covariate shift^26^. Next, a Max Pooling layer with a size of $\:2\times\:2$ is applied to reduce the spatial resolution of the feature maps while retaining the most salient information, thus enhancing computational efficiency and providing spatial invariance.

A Dropout layer with a 20% rate is introduced to mitigate overfitting by randomly setting a fraction of the input units to zero during training^27^. This prevents the network from relying too heavily on specific neurons and encourages redundancy in feature representation.

The second convolutional block follows a similar structure but employs 64 filters and a $\:3\times\:3$ kernel size, allowing the network to detect finer spatial patterns. It is again followed by batch normalization, pooling, and a higher dropout rate of 30%, reflecting the increased model capacity at this stage.

Rather than using a flattening operation—which can lead to overfitting and high parameter counts—a Global Average Pooling (GAP) layer is employed. GAP computes the average of each feature map, effectively reducing the dimensionality while preserving important spatial information. Mathematically:

$$\:{Z}_{k}=\:\frac{1}{H\:.\:W}\:\sum\:_{i=1}^{H}\sum\:_{j=1}^{W}{Y}_{i,j,k}$$

Where $\:H$ and $\:W$ are the height and width of the feature map and $\:{Z}_{k}$ is the output for feature map $\:k$. GAP also encourages the model to focus on the entire activation map, thereby improving generalization^28^.

The output of the GAP layer is passed through a fully connected (dense) layer with 128 neurons and ReLU activation, enabling the model to learn non-linear combinations of high-level features. Another 30% dropout layer is used at this stage for further regularization.

The final layer is a softmax-activated dense layer, with the number of neurons equal to the number of stratigraphic zone classes. The softmax function converts the output logits into a probability distribution:

$$\:softmax\:\left({Z}_{i}\right)=\:\frac{{e}^{{z}_{i}}}{{\sum\:}_{j=1}^{K}{e}^{{z}_{j}}}$$

Where $\:{Z}_{i}$ is the input to the $\:{i}^{th}$ output neuron and $\:K$ is the total number of classes. The zone with the highest predicted probability is selected as the model’s output. This architecture is trained using categorical cross entropy loss, defined as:

$$\:\:L=\:\sum\:_{i=1}^{HK}{y}_{i}\text{l}\text{o}\text{g}\left({\widehat{y}}_{i}\right)$$

Where $\:{y}_{i}$ us the true label and $\:{\widehat{y}}_{i}$ is the predicted probability.

The training of the Convolutional Neural Network (CNN) was designed to ensure effective learning while minimizing overfitting. The Adam optimizer was selected for model training due to its adaptive learning rate capabilities and computational efficiency^29^. Adam optimizer combines the advantages of two widely used extensions of stochastic gradient descent: Adaptive Gradient algorithm (AdaGrad), which works well with sparse gradients, and Root Mean Square Propagation (RMSProp), which handles non-stationary objectives.

To enhance generalization and optimize training dynamics, a set of Keras callbacks was employed:

- *Early Stopping*: Training was halted if the validation loss did not improve for 10 consecutive epochs (*patience = 10*). This prevents overfitting by terminating training once performance plateaus, avoiding unnecessary epochs and reducing the risk of memorizing noise in the training data^30^.
- *Model Checkpointing*: At each epoch, the model state with the lowest validation loss was saved to disk. This ensures that the final deployed model reflects the best generalization performance, not merely the last epoch.
- *Adaptive learning rate (ReduceLROnPlateau)*: The learning rate was automatically reduced by a factor of 0.5 if the validation loss showed no improvement for 5 consecutive epochs. This mechanism enables finer convergence in later training stages by decreasing the learning step size, allowing the model to escape shallow local minima and refine its predictions.

The model was trained for a maximum of 200 epochs, with a batch size of 16, balancing memory efficiency and gradient estimate stability^31^. To ensure robust model evaluation, 20% of the training data was withheld for validation during each epoch. This allowed real-time monitoring of the model’s generalization performance.

### Model evaluation

To evaluate the performance of the proposed CNN model, multiple assessment metrics were utilized to ensure a comprehensive and reliable measure of its classification capability. A confusion matrix was used to visualize the distribution of correct and incorrect predictions across stratigraphic zones, enabling the identification of specific misclassifications and assessing the model’s ability to differentiate between closely related depositional units.

Additionally, overall accuracy—calculated as the ratio of correctly predicted zones to the total number of predictions—was adopted as a global performance metric during both training and testing phases. The accuracy metric is defined by the following equation:

$$\:Accuracy=\:\frac{TP+TN}{TP+TN+FP+FN}$$

Where TP (True Positive) are samples from positive class that are correctly predicted as positive, TN (True Negative) are the samples from negative class that correctly predicted as negative, FP (False Positive) are samples from the negative class that are incorrectly predicted as positive and FN (False Negative) are the samples from the positive class that predicated as negative.

Beyond accuracy, a classification report was generated to include precision, recall, and F1-score for each class, offering deeper insight into model performance, especially for imbalanced datasets^32^. The following equations define the mathematical expressions for these evaluation metrics:

$$\:Precision=\:\frac{TP}{TP+FP}$$

$$\:Recall=\:\frac{TP}{TP+FN}$$

$$\:F1\_score=2*\:\frac{Precision*Recall}{Precision+Recall}\:$$

These metrics ensured a balanced evaluation and helped detect any potential bias toward dominant classes.

Feature importance was quantified using a permutation-based approach, which measures the decrease in model accuracy when a specific feature’s values are randomly shuffled. This process disrupts the relationship between that feature and the target variable while keeping the model and all other features fixed. The average accuracy drop over 100 repetitions was used as an indicator of feature relevance. This method provides a robust, model-agnostic estimate of each input feature’s contribution to the CNN’s predictive performance^33–35^.

### Implementation workflow

The implementation of the trained CNN model on a blind well begins with the collection of the required input features: *GR*,* NPHI*,* RHOB*, *PE* and Facies logs. Additional features are derived for each well, including the True Vertical Depth Subsea (*TVDSS*), computed using the well deviation survey and the minimum curvature method, and three spatial distance features calculated from reference points surrounding the study area. These features are extracted at the start and end depths of the Lower Bahariya member for each blind well.

Following feature extraction, the blind well data is normalized using the same min-max scaling parameters derived from the training dataset, ensuring consistency in the input domain across both training and prediction phases. A sliding window approach is employed to segment the Lower Bahariya interval into multiple depth windows. Each window is interpolated (stretched or compressed) to a fixed size of 64 depth samples, maintaining the stratigraphic resolution regardless of the original window thickness.

For each rescaled window, nine 64 × 8 grayscale images are generated—one for each input feature. These images simulate stratigraphic depth sequences and are formatted into 3D tensors that are then passed through the trained CNN model. The model returns a probability distribution across the zone classes for each window, from which the zone with the highest predicted probability is assigned to that window. The window is then shifted downward by a predefined step size, and the process is repeated until the entire interval is evaluated.

To account for variability in zone thickness and ensure robustness, this process is executed for a range of window sizes. The output of each prediction includes the start and end depth of the window, the predicted zone, the window size used, and the corresponding prediction confidence (match percentage). All predictions are compiled into a result table.

To refine the output and improve reliability, two post-processing filters are applied:

- Thickness Filter: Predicted zones falling outside their known stratigraphic thickness range are discarded.
- Sequence Filter: The final zonation must follow the expected vertical stratigraphic order. Only the most confident prediction for each zone is retained, and any instances of that zone appearing out of sequence (e.g., below a lower zone or above an upper one) are excluded.

**Fig. 7:**
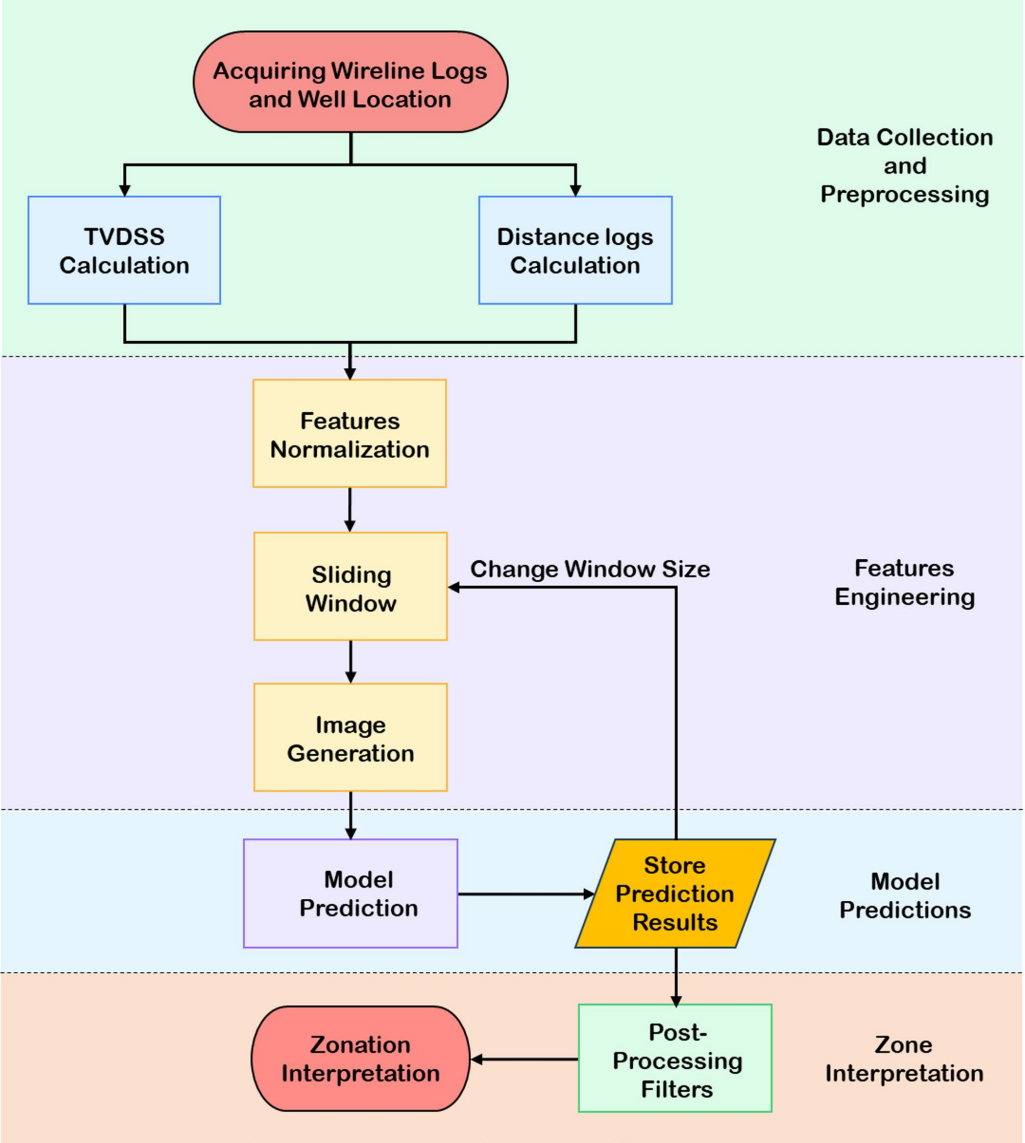
Implementation workflow for applying the trained CNN model on a blind well.

Finally, the most appropriate window size and the optimum mid-depth for each zone are interpreted from the summary charts and visualizations, allowing accurate zonal identification in the blind well. The overall implementation workflow of the CNN-based zonation approach is illustrated in Fig. 7.

## Results and discussion

To monitor the model’s learning dynamics, accuracy and loss metrics were tracked across 120 training epochs for both training and validation datasets.

**Fig. 8:**
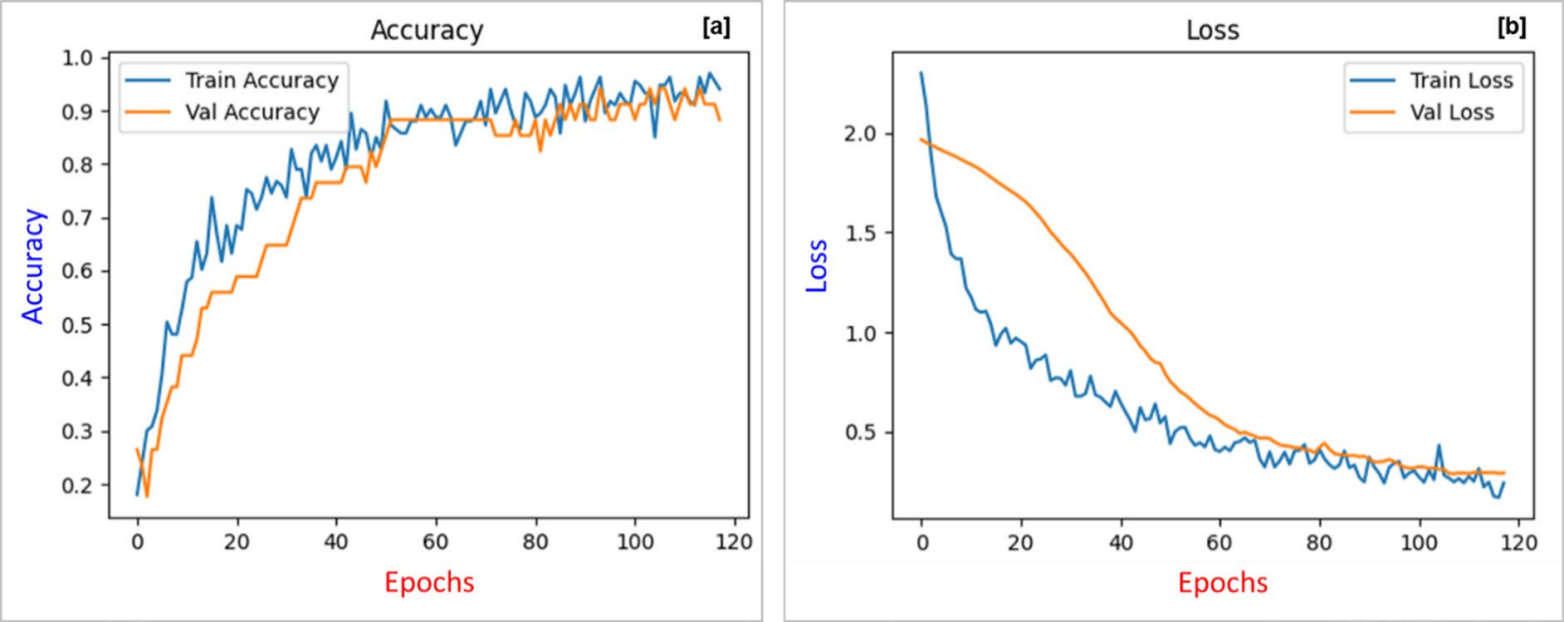
Training performance of the proposed CNN model: (**a**) Training and validation accuracy showing progressive improvement and convergence over epochs. (**b**) Training and validation loss curves illustrating steady decline and convergence, indicating effective learning and generalization.

Figure 8a illustrates the accuracy curves over training epochs. Both training and validation accuracies exhibit a steady upward trend, surpassing 90% after epoch 60. The minimal gap between the two curves suggests robust generalization and negligible overfitting. The performance plateau observed after epoch 80 indicates the model’s convergence and learning stabilization.

Figure 8b presents the corresponding loss curves. The training loss rapidly declines in the initial epochs and gradually stabilizes, while the validation loss follows a similar decreasing trend with mild fluctuations due to variability in the validation set. The convergence of both accuracy and loss metrics confirms the model’s capability to capture essential patterns in the input features and effectively differentiate stratigraphic zones.

The CNN model demonstrated strong generalization capabilities on unseen data, achieving an overall testing accuracy of 94.1%. The classification report, shown in Table 5, highlights the detailed performance for each stratigraphic zone:

**Table 5:** Classification report summarizing the CNN model’s performance on the test set.

Zone	Precision	Recall	F1-score	Support
Cycle A	1.00	1.00	1.00	4
Upper Sand Unit (USU)	0.80	0.80	0.80	5
Shale Barrier 1	1.00	1.00	1.00	4
Middle Sand Unit (MSU)	0.75	0.75	0.75	4
Shale Barrier 2	1.00	1.00	1.00	4
Lower Sand Unit (LSU)	1.00	1.00	1.00	4
Tidal flat base	1.00	1.00	1.00	3

The macro average for precision, recall, and F1-score reached 0.94, 0.94, and 0.94, respectively, while the weighted averages were 0.93, 0.93, and 0.93. These results underscore the model’s consistent classification performance across all stratigraphic zones.

**Fig. 9:**
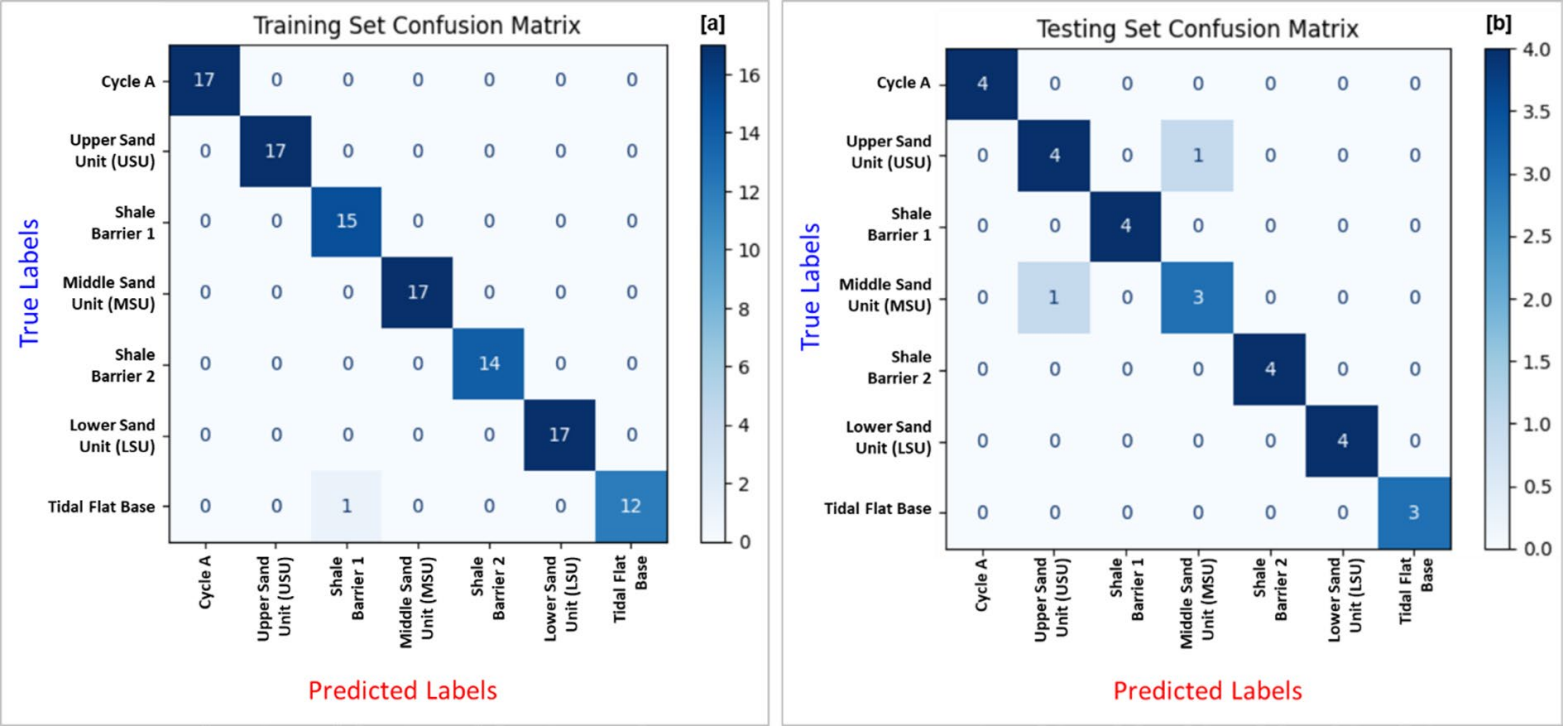
Confusion matrices illustrating the classification performance of the CNN model: (**a**) Training set confusion matrix showing high prediction accuracy across all zone classes with minimal misclassifications, and (**b**) Testing set confusion matrix demonstrating the model’s generalization ability on unseen data, with overall strong agreement between predicted and true labels.

Figure 9 presents the confusion matrices for both the training and testing datasets. In the training set matrix (Fig. 9a), the model achieved nearly perfect classification with negligible misclassifications, indicating effective learning of the training data patterns. In the testing set matrix (Fig. 9b), the model maintained strong performance, accurately predicting most stratigraphic zones. Minor misclassifications were observed, particularly between lithologically similar or adjacent zones such as the Middle Sand Unit and Shale Barrier. Nevertheless, the model successfully captured the complexity of the depositional environment with reliable accuracy on unseen data.

To quantify the relative contribution of each input feature to the CNN classification model, permutation feature importance analysis was performed. In this technique, each feature was randomly permuted multiple times while keeping the others unchanged, and the resulting decrease in classification accuracy was measured. The average accuracy drop across 100 permutations was used as an importance score for each feature.

**Fig. 10:**
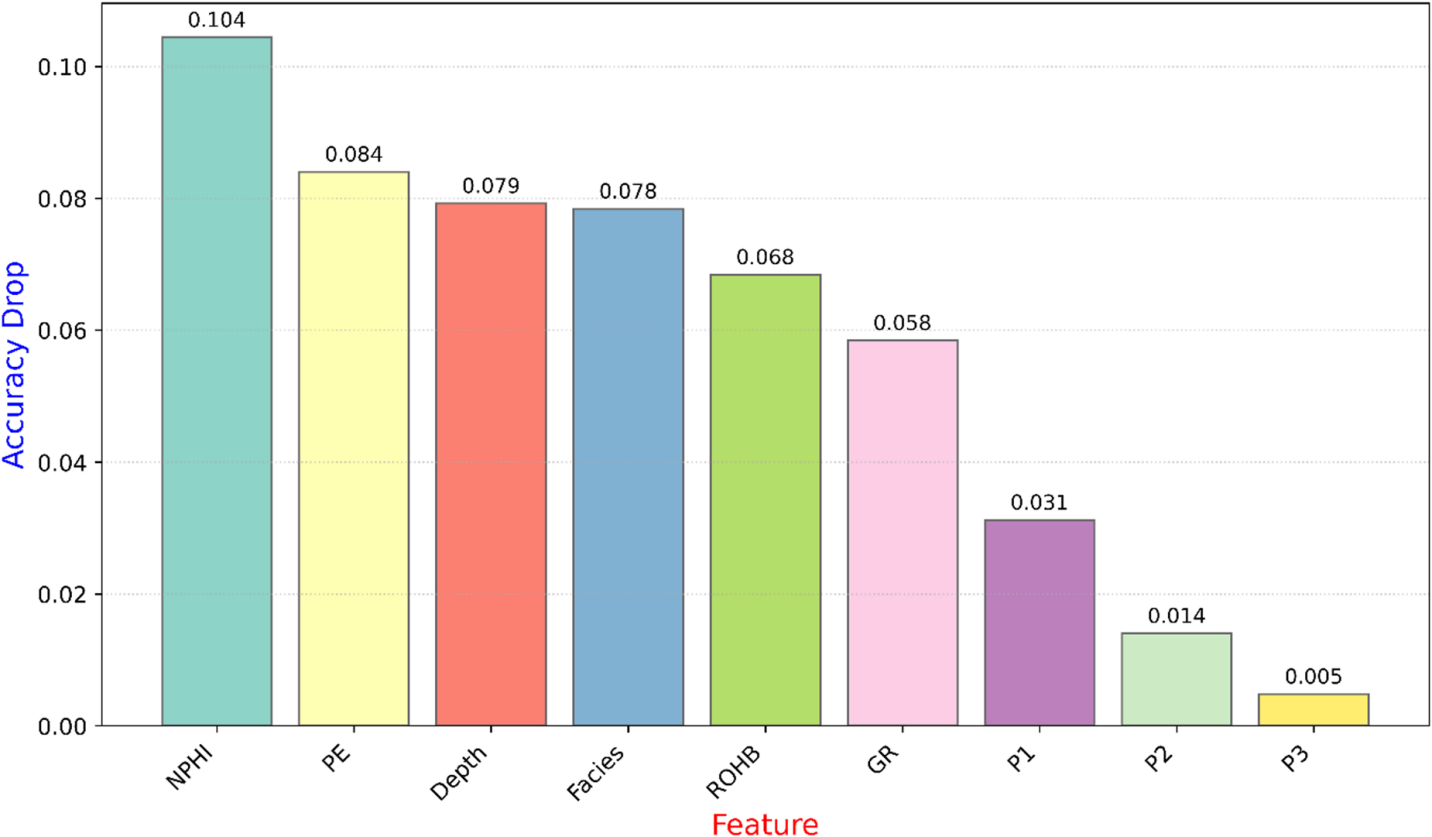
Permutation feature importance derived from the convolutional neural network (CNN) classification model. Each bar represents the mean decrease in model accuracy when the corresponding feature is randomly permuted, averaged over 100 repetitions. Features causing higher accuracy drops are considered more influential on the model’s predictive performance.

The feature importance results (Fig. 10) reveal that the CNN model predominantly relies on petrophysical features with strong lithological sensitivity. *NPHI* and *PE* exhibit the greatest impact on model performance, emphasizing their effectiveness in capturing compositional variations and lithofacies transitions across the studied interval. *Depth* and *facies* follow closely, indicating that both stratigraphic position and categorical facies representation significantly aid the network’s ability to delineate stratigraphic zones. The *GR* and *ROHB* logs contribute moderately, reflecting their supplementary role in distinguishing shale-sand alternations. Meanwhile, the *P1*, *P2*, and *P3* distance logs, although less influential individually, remain important as they encode the relative spatial and stratigraphic positioning of wells within the field. These position-based features allow the model to account for lateral facies variations and depositional trends, ensuring that predictions remain consistent with the regional stratigraphic framework.

The developed CNN model was applied to a blind test well (Shahd SE-26) to evaluate its real-world applicability in stratigraphic zonation. The input features for the well—Gamma Ray (GR), Neutron Porosity (NPHI), Bulk Density (RHOB), and Photoelectric Effect (PE), along with the calculated TVDSS and distance logs—were first collected and pre-processed. These features were then standardized using the same normalization parameters derived from the training dataset to ensure consistency in input distribution.

To account for variable zone thicknesses, a sliding window approach was employed. Window size, representing the number of samples per segment, ranged from 5 to 200 samples (equivalent to 2.5 ft to 100 ft) in increments of 5 samples per iteration. This strategy allowed comprehensive coverage of all stratigraphic zone thicknesses encountered during training. For each window interval, the model predicted the most likely stratigraphic zone label along with a corresponding match percentage indicating classification confidence.

**Fig. 11:**
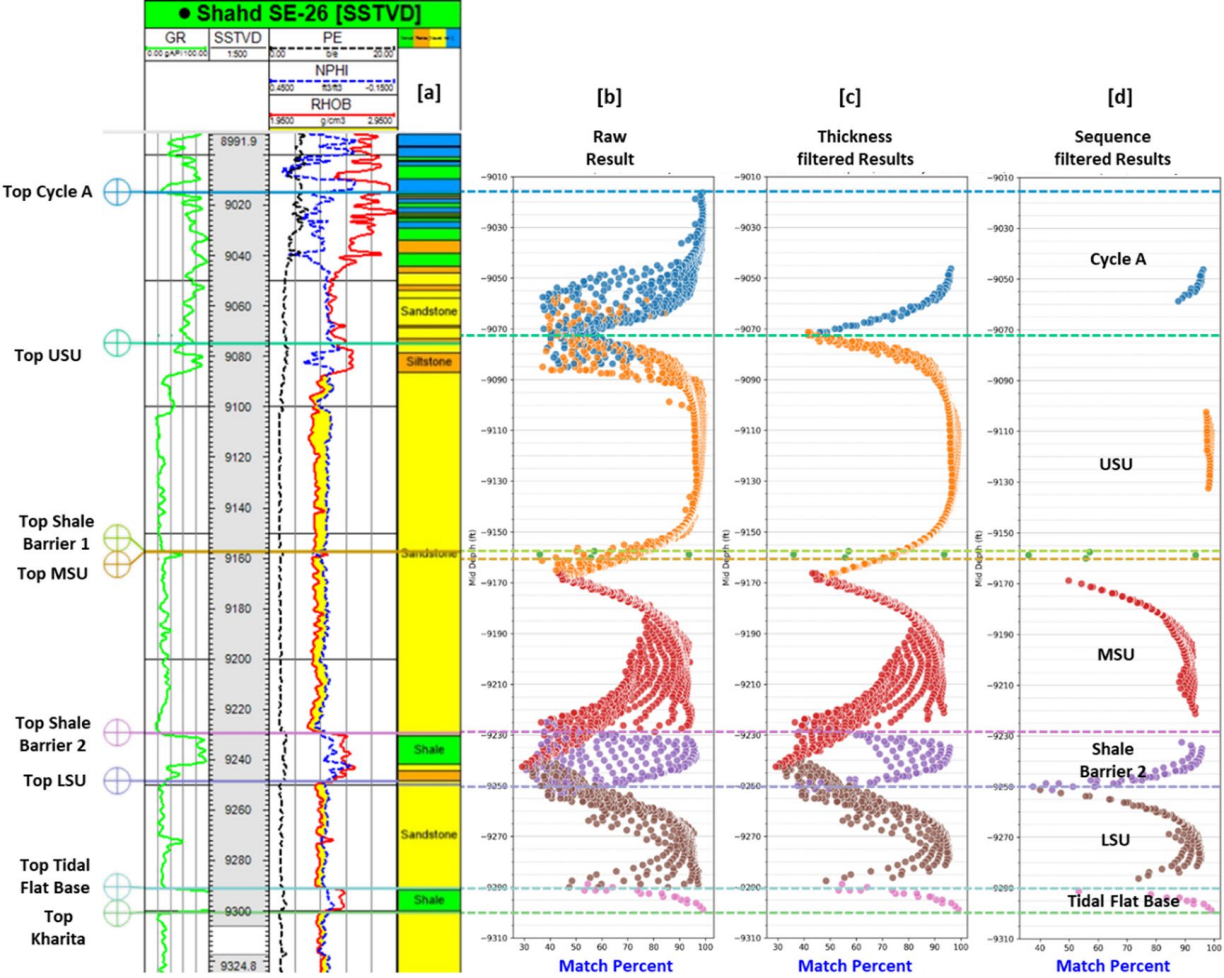
Comparison of expert-interpreted zonation and model-based predictions for the Shahd SE-26 well. (**a**) shows the traditional zonation derived from wireline log interpretation. Panels to the right illustrate the machine learning model predictions at three stages: (**b**) raw predictions for all window sizes, (**c**) results after applying the thickness filter, and (**d**) final filtered predictions after enforcing sequence consistency.

Figure 11a presents the expert-interpreted zonation for the Shahd SE-26 well, derived from traditional analysis of log responses. In comparison, Fig. 11b–d illustrate the results obtained from the machine learning model. In these figures, the Y-axis denotes the mid-depth of each predicted interval, while the X-axis represents the model’s prediction match percentage.

Figure 11b shows the raw prediction output across all evaluated window sizes, capturing the full range of model-generated zonation candidates. To enhance interpretability and geological plausibility, a two-stage post-processing approach was applied:

1. Thickness Filter: Predictions that fall outside the established geological thickness ranges for each stratigraphic zone (as detailed in Table 2) were excluded. The refined results after applying this constraint are depicted in Fig. 11c, showing a more geologically reasonable distribution.
2. Sequence Filter: A stratigraphic consistency check was implemented to preserve the expected vertical ordering of zones. For each predicted zone, the most representative interval was selected based on the highest match percentage and recurrence across multiple window sizes. Any predictions that violated the natural depositional sequence—such as a deeper zone appearing above a shallower one—were removed. The final, geologically consistent zonation output is shown in Fig. 11d.

A summary of the most representative predicted intervals for each zone—based on their depth range, match percentage, and window size—is presented in Table 6. The match percent refers to the model’s classification confidence derived from the Softmax output layer of the CNN, which converts the raw prediction scores into normalized probabilities across all possible zones. Each interval’s match percentage corresponds to the highest Softmax probability associated with the predicted class, representing how confidently the model assigns that interval to a specific stratigraphic zone. Higher match percentages therefore indicate stronger model certainty in the classification decision.

The model’s blind prediction results demonstrated high consistency with expected geological patterns, validating its robustness for application in wells lacking prior stratigraphic interpretation. Geological consistency was further evaluated through expert comparison with manually interpreted correlations and reference to the biostratigraphic conceptual model. The predicted zones showed strong agreement with established stratigraphic markers and preserved lateral facies transitions aligned with the regional depositional framework, confirming the geological reliability of the model outputs.

**Table 6:** Final predicted stratigraphic intervals for the blind test well, representing the most probable match for each zone.

Zone	Start depth (ft.)	End depth (ft.)	Mid depth (ft.)	Thickness (ft.)	Match percent
Cycle A	− 9015.26	− 9075.26	− 9045.26	60	0.96
USU	− 9078.26	− 9158.26	− 9118.26	80	0.99
Shale Barrier 1	− 9157.78	− 9159.76	− 9158.76	2.5	0.93
MSU	− 9160.26	− 9230.26	− 9195.26	70	0.93
Shale Barrier 2	− 9230.26	− 9250.26	− 9240.26	20	0.95
LSU	− 9148.76	− 9288.76	− 9168.76	40	0.95
Base tidal flat	− 9289.76	− 9299.76	− 9254.76	10	0.99

**Fig. 12:**
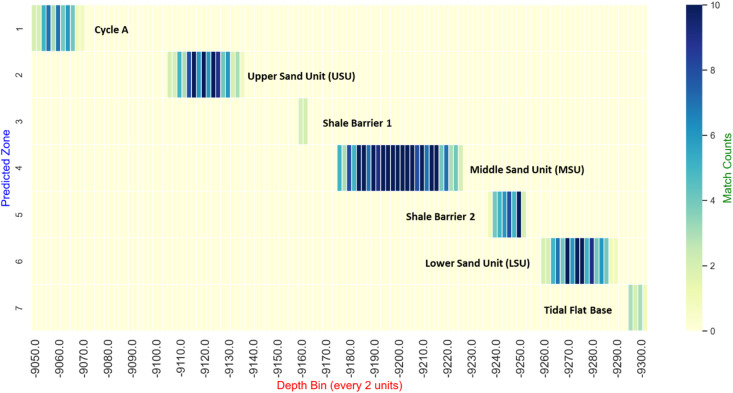
Heatmap showing the distribution of prediction support (match counts) for each stratigraphic zone across depth bins. Darker colors indicate higher support and therefore greater model confidence, while lighter colors represent lower confidence predictions. Depth bins are binned every 2 units, and each row corresponds to a predicted stratigraphic zone.

Figure 12 presents a heatmap of prediction support across depth bins for each predicted stratigraphic zone. The color intensity corresponds to the number of samples supporting a given zone at each depth, effectively visualizing the confidence of the model predictions. Zones such as the Middle Sand Unit (MSU) and Upper Sand Unit (USU) show higher support counts (darker colors), indicating strong model agreement, whereas transitional or barrier layers exhibit lighter colors, reflecting lower confidence. This visualization provides a clear and intuitive representation of prediction uncertainty along the wellbore, complementing the quantitative “match percentage” metric.

The final results produced by the machine learning model show a strong agreement with the expert interpretation, particularly within the Cycle A, Upper Sand Unit (USU), and Tidal Flat Base. Notably, despite the very limited thickness of Shale Barrier 1—which was considered pinched out in the expert interpretation—the CNN model successfully identified its presence. The predicted location of this zone aligns well with the stratigraphy of the adjacent overlying and underlying units, demonstrating the model’s ability to detect subtle geological features.

## Conclusions

This study demonstrates the effective application of convolutional neural networks (CNNs) for automating stratigraphic zonation within the Lower Bahariya member. By transforming well log data into 2D image representations and training the model on a carefully pre-processed and balanced dataset, the CNN was able to accurately learn and differentiate complex depositional patterns across multiple stratigraphic units. The model achieved a high testing accuracy of 94.1% and delivered strong performance across all evaluation metrics, including precision, recall, and F1-score—highlighting its ability to generalize to unseen data.

The workflow also included a robust implementation strategy on a blind test well using a sliding window approach and post-processing filters that enforced geological constraints such as thickness consistency and depositional sequence. The results showed high agreement with expert interpretations, even successfully identifying subtle features like thin shale barriers that are often overlooked in manual correlation.

Overall, the integration of machine learning in this study provides a reliable, objective, and scalable solution for stratigraphic interpretation. It addresses key challenges in correlating sand channels and shale barriers, which directly impact reservoir connectivity and simulation performance. The proposed methodology can be extended to other fields and formations, paving the way for more data-driven and consistent subsurface interpretations in future reservoir studies.

## Data Availability

The data supporting the results of this study was obtained from the Egyptian General Petroleum Corporation. Data are, however, available from the corresponding author upon reasonable request and with permission of the Egyptian General Petroleum Corporation.In addition, the preprocessing code, trained model weights, and example scripts used in this study can be shared by the corresponding author upon reasonable request, subject to data confidentiality agreements. This ensures transparency and reproducibility of the workflow while respecting data ownership and confidentiality policies.
